# A clinical procedures curriculum for undergraduate medical students: the eight-year history of a third-year immersive experience

**DOI:** 10.3402/meo.v21.29486

**Published:** 2016-05-23

**Authors:** Laura Thompson, Matthew Exline, Cynthia G. Leung, David P. Way, Daniel Clinchot, David P. Bahner, Sorabh Khandelwal

**Affiliations:** 1 Department of Emergency Medicine, The Ohio State University College of Medicine, Columbus, OH, USA; 2 Department of Internal Medicine, The Ohio State University College of Medicine Columbus, OH, USA; 3 Department of Physical Medicine & Rehabilitation, The Ohio State University College of Medicine, Columbus, OH, USA

**Keywords:** education, undergraduate medical, clinical competence, bloodless medical and surgical procedures, life support care, diagnostic techniques and procedures

## Abstract

**Background:**

Procedural skills training is a critical component of medical education, but is often lacking in standard clinical curricula. We describe a unique immersive procedural skills curriculum for medical students, designed and taught primarily by emergency medicine faculty at The Ohio State University College of Medicine.

**Objectives:**

The primary educational objective of this program was to formally introduce medical students to clinical procedures thought to be important for success in residency. The immersion strategy (teaching numerous procedures over a 7-day period) was intended to complement the student's education on third-year core clinical clerkships.

**Program design:**

The course introduced 27 skills over 7 days. Teaching and learning methods included lecture, prereading, videos, task trainers, peer teaching, and procedures practice on cadavers. In year 4 of the program, a peer-team teaching model was adopted. We analyzed program evaluation data over time.

**Impact:**

Students valued the selection of procedures covered by the course and felt that it helped prepare them for residency (97%). The highest rated activities were the cadaver lab and the advanced cardiac life support (97 and 93% positive endorsement, respectively). Lectures were less well received (73% positive endorsement), but improved over time. The transition to peer-team teaching resulted in improved student ratings of course activities (*p*<0.001).

**Conclusion:**

A dedicated procedural skills curriculum successfully supplemented the training medical students received in the clinical setting. Students appreciated hands-on activities and practice. The peer-teaching model improved course evaluations by students, which implies that this was an effective teaching method for adult learners. This course was recently expanded and restructured to place the learning closer to the clinical settings in which skills are applied.

Until recently, medical schools relied primarily on an apprenticeship model, teaching common clinical procedures through observation, trial, and demonstration; see one, do one, teach one (1). Unfortunately, the apprenticeship model does not provide sufficient experience and practice with procedural skills to adequately prepare students for residency. The apprenticeship model is highly variable and greatly dependent on patient case-mix, care setting, and other clerkship circumstances such as competition with higher level learners for learning opportunities. Studies have shown that students do not get sufficient opportunity to practice or perform basic clinical procedures such as venipuncture, arterial puncture, or insertion of venous catheters or nasogastric (NG) tubes. Furthermore, most students never have the opportunity to observe, let alone learn and practice, more advanced procedures such as lumbar puncture, thoracentesis, or paracentesis (2–6). Additional studies have shown that simple observation of procedures does little to help medical students talk to patients about clinical procedures or confidence to perform them (3, 5–8).

More recently, medical schools have adopted formal procedural skills training curricula. One study, comparing schools with traditional curricula to those with formal skills training, found that the latter contributed to higher scores on written skill assessments. Additionally, students with formal procedural training maintained skills performance superiority over subsequent years of medical school (9). Several studies of new clinical skills curricula have demonstrated significant improvements in medical student confidence after implementation of formal procedural skills courses (8, 10, 11).

Literature on how much procedural skills training is needed at the undergraduate medical school level has yielded mixed results. One study demonstrated that just one training session covering several clinical procedures produced significant improvements in medical student's competence and confidence (12). Another study demonstrated that increases in the frequency and quality of feedback involving only one procedure (NG tube placement) contributed to improving medical students’ ‘global procedural performance’ (13). Participation in a formal procedures course during medical school has been associated with higher self-assessed competency among those entering residency (7). Collectively, these studies suggest that medical students need a formal procedural skills program to improve their knowledge about and opportunities to practice clinical procedures. Formal procedural skills programs should be longitudinal in nature to bridge the transition from undergraduate medical education (UGME) into graduate medical education (GME).

Prior to 2006, all procedural training at our medical school was conducted through the traditional clinical clerkship model. An Introduction to Clinical Medicine (ICM) course offered some basic skills training prior to the clinical clerkships, but most training was dispersed throughout the clinical curriculum. Consequently, in the decade prior to our procedures course, our medical students routinely rated ‘the opportunity to learn and practice clinical procedures’ as one of the most significant deficits of their medical education. This finding was consistent across multiple evaluations, including the AAMC Graduation Questionnaire (GQ) (14), and our own clinical curriculum evaluation, graduate follow-up, and residency director surveys. To address student concerns about the procedures deficit to their education, we introduced the Clinical Skills Immersion Experience (CSIE) into our clinical curriculum.

## Conceptual framework

The primary purpose of the CSIE was to provide our medical students with first-hand, hands-on experience with the clinical procedures they see during their clinical rotations. These procedures, we believe, were far too advanced for competency-based or mastery learning, particularly with the time allocated to this program. However, we wanted students to sufficiently understand the impact of procedures so that they felt comfortable discussing them with the patients under their care. Furthermore, through the immersive experience, we covered the most common procedures so that as a student progresses into residency, he/she will be comfortable with patient communication regarding the procedures for which they will most likely be responsible.

## Objective

To provide more standardized clinical procedures training, we instituted a formal procedural skills course in 2006, called the Clinical Skills Immersion Experience. The 7-day course provided 60 h of formal training in 27 routine procedural skills through a variety of teaching methods. The goals of the course were twofold. First we wanted to provide sufficient experience and understanding of the procedures commonly seen during clerkships so that students are capable of discussing procedures with their patients. Second, we wanted to introduce students to the procedures that they will eventually be responsible for as residents through hands-on training and practice. The purpose of this paper is to describe the development, implementation, and progression of the course over an 8-year period. To this end, we used the outline for disseminating curriculum development proposed by Reznich and Anderson (15).

## Curriculum design

### Development

The CSIE was designed to introduce key clinical procedures that would complement the student's education during their third-year core clinical clerkships: Family and Internal Medicine, Neurology, Obstetrics & Gynecology, Pediatrics, Psychiatry, and Surgery. Key procedures were those thought to be necessary for success in residency programs and were selected on the basis of ‘need’ as defined by a faculty curriculum taskforce. Besides the data sources already mentioned, the taskforce used student focus groups, and interviews with clerkship and residency program directors for their needs assessment.

### Curriculum

The resulting 7-day course was tacked on to a 6-week core clerkship in a third-year curriculum comprised mostly of 4- and 8-week clerkships. Accordingly, the CSIE was taught six times per year. The course was designed to be hands-on and made extensive use of a new Clinical Skills Education and Assessment Center (CSEAC). The course covered both cognitive and motor skill aspects of key procedures. The teaching and learning methods (TLMs) included lectures, demonstrations, readings, videos, simulations, a cadaver lab, and online learning modules.

### Content material

Key procedures covered in the CSIE course are listed in Table 1 along with the methods used to teach them. Students were given primary and secondary learning objectives for each of the 27 skills addressed in this course. They were also assigned to small groups for the hands-on simulations and labs. Procedure skills were inventoried and assessed using checklists. Initially, checklists and assessments were completed by faculty; however, peers served as evaluators after a peer-team teaching model was adopted in 2010.

**Table 1:** List of 27 key procedures taught to third-year medical students during a 7-day Clinical Skills Immersion Experience (CSIE) course at The Ohio State University College of Medicine with methods used for teaching

	Lectures or demos	Independent learning: reading, videos, or online modules	Hands-on practice with lo- or hi-fidelity simulations	Cadaver laboratory
Advanced cardiac life support (ACLS)	•		•	
Advanced trauma life support (ATLS)	•			
Anoscopy		•	•	
Arterial cannulation				
Airway management/intubation		•	•	•
Central line placement		•	•	
Colonoscopy	•	•		
Cricothyrotomy		•	•	
Emergency medical services	•		•	
Emergency transcutaneous pacing		•	•	
Endoscopy	•	•		
Evidence based medicine	•	Journal Club		
Hemodialysis	•	•		
Incision & drainage of abscesses	•	•	•	
Kidney biopsy		•		•
Lumbar puncture	•	•	•	•
MRI/CT interpretation	•			
Paracentesis	•	•		•
Pericardiocentesis	•	•	•	•
Skin biopsy	•	•	•	
Splinting and casting	•	•	•	
Swan-Ganz catheter		•		
Thoracentesis		•	•	
Tube thoracostomy		•		•
Ultrasound	•		•	
Vent management	•		•	
Wound management & laceration repair	•	•	•	

### Peer-team teaching model

In the peer-team teaching model, students were assigned to groups of six students for the entire course. The peer-teams met for 1 h at the beginning of each day to review the objectives and learning materials for the day. Peer-teams were encouraged to work together and were tested together during the final examination.

### Logistics

An example of 1 day of the course is presented here. The procedures covered during this day included: airway and ventilator management, review of advanced cardiac life support (ACLS), central venous catheter placement, lumbar puncture, and cricothyrotomy. Students prepared by: viewing videos from the *New England Journal of Medicine*'s website on Central Venous Catheterization and Positive Pressure Ventilation with a Face Mask and Bag-Valve Device; reviewing the ACLS website and basic airway checklist; and reading about central line placement, ventilator management, and airway management for trauma patients. During the day, students performed ACLS simulations, and practiced central venous catheter placement, basic airway and ventilator management, lumbar punctures, and cricothyrotomy in the CSEAC. Table 2 shows the course syllabus for theweek.

**Table 2:** Seven-day syllabus for Clinical Skills Immersion Experience (CSIE) course at The Ohio State University College of Medicine

	Thursday	Friday	Monday	Tuesday	Wednesday	Thursday	Friday
**7–8 am** **8–9 am** **9–10 am** **10–11 am** **11–noon** **12–1 pm** **1–2 pm** **2–3 pm** **3–4 pm** ***4–5 pm*** *Homework*	7:45a–8:30a **Orientation** 8:30a–9:30a Lecture: **Cardiac** **Resuscitations** 9:30a–10:30a Lecture: **Suturing** 10:30p–1:00p **Suturing** **Workshop** Lecture: **Emergency Medical Services (EMS)** 2:00p–3:30p **EMS Practical** 3:30p–4:30p **Closing topics and weekend journal club assignment**	*Peer-Team* Meetings 8:00a–11:00a **Practice Cardiac** **Resuscitations (ACLS)** ***Anoscopy*** **Central Line** **Placement** **Splinting/Casting** ***Knot Tying*** **Complete on-line** **learning modules** **on Colonoscopy** ***EGD &*** ***Kidney Biopsy*** *Break* Lecture: **Colonoscopy** 1–2:30p Lecture: **Adv Trauma Life Support** 2:30p–3:30p Lecture: **Vent** **Management** Read **Mammography PPT**	*Peer-Team* Meetings 8:00a–11:00a **Airway** **Simulation** **Practice Cardiac** **Resuscitations** **(Dysrhythmias)** **Central Line** **Placement** **Vent** **Management** **Lumbar** **Puncture** **Cricothyrotomy** *Break* 11:30a–1:30p **Journal Club** **Presentations** 1:30p–4:30p **Ultrasound** Read **Swan-Ganz** ***Hemodynamic Monitoring***	*Peer-Team* Meetings 8:00a–9:30a **Anatomy** **Review** Grps 4–6 9:30a–11:00a **Anatomy** **Review** Grps 1–3 *Break* 12:00p–4:00p **Paracentesis** **Pericardiocentesis** **Thoracentesis** **Lumbar Puncture**	*Peer-Team* Meetings ***Chest Tube*** Grps 1–3 ***Chest Tube*** Grps 4–6 10:00a–10:30a **Intraosseous Infusion** Grps 4–6 10:30a–11:00a **Intraosseous Infusion** Grps 1–3 *Break* Lecture: **Hemodialysis** 1:00p–1:45p **Airway** on a Cadaver Grps 1–3 1:45p–2:30p **Airway** on a Cadaver Grps 4–6	*Peer-Team* Meetings 8:00a–11:00a **Arterial Catheter** **I&D Abscess** **Skin Biopsy** **Peripheral IV** **Dialysis** **Hemodialysis** *Break* 12:00p–3:00p ***Approach*** **to Reading CT/MRI** *Time for studying or ‘extra practice’*	8:30a–11a **Written Exam** **11a-noon Course Evaluation**

Topics or activities are listed in boldface.

## Program impact

### Course evaluations

The course evaluation was a 24-item questionnaire adapted from the standard course evaluations used by our college of medicine for all its programs and courses. The adaptations asked students to rate the utility of specific course elements (Table 3). Students rated features of the course using a traditional 5-point Likert Scale. Two open-ended questions asked students to comment on the course's strengths and weaknesses. Responses to the evaluation questionnaire were aggregated into percentage of positive endorsement (ratings of 4 or 5) and non-positive endorsement (ratings of 1–3) and reported by month and year in which the course was taken (August, October, December, February, April, or June; 2006–07 through 2013–14).

**Table 3:** Twenty-four item course evaluation questionnaire for Clinical Skills Immersion Experience (CSIE) course at The Ohio State University College of Medicine

The on-line learning modules were well organized.
Assigned readings/didactic materials were appropriate for third-year medical students.
Assigned readings/didactic materials helped me understand the procedures/skills.
Assigned readings/videos/materials helped prepare me to perform the procedure/skills.
The faculty instructors were enthusiastic.
The faculty instructors were well prepared.
The Clinical Skills Education and Assessment Center (CSEAC) staff was helpful.
The skills/procedures taught were appropriate for third-year medical students.
The checklists were helpful in helping me keep track of my procedures learning and performance.
The Anatomy Review Day helped me better understand and perform the procedures.
Working on cadavers was beneficial to my understanding the procedures.
The workshops/lectures provided me sufficient knowledge and skills to learn ACLS/Code.
The workshops/lectures provided me sufficient knowledge and skills to learn Ultrasound.
The workshops/lectures provided me sufficient knowledge and skills for Journal Club.
The workshops/lectures provided me sufficient knowledge and skills to learn an approach to reading and interpreting MRI/CT.
The workshops/lectures provided me sufficient knowledge and skills to learn Hemodialysis.
The workshops/lectures provided me sufficient knowledge and skills to learn ATLS.
The workshops/lectures provided me sufficient knowledge and skills to learn EMS.
The workshops/lectures provided me sufficient knowledge and skills to learn Suturing.
Feedback on my performance during the sessions was helpful.
Overall, the CSIE was educationally worthwhile.
I feel better prepared for future clinical clerkships.
I think this course will benefit me in my PGY-1 year.
I think the material covered in the CSIE was well reflected in the written examination.

Overall student ratings of the CSIE course were relatively high. Almost all (97.7%) students positively endorsed the statement: ‘the CSIE was educationally worthwhile’. Additionally, almost all learners positively endorsed the statements: ‘I feel better prepared for future clinical clerkships’ (96.1%) and ‘I think this course helped prepare me for residency’ (93.4%). Positive ratings of student preparation for residency were particularly salient when we consider that all students, including those pursuing specialties with low procedures involvement, responded positively to this item.

**Fig. 1:**
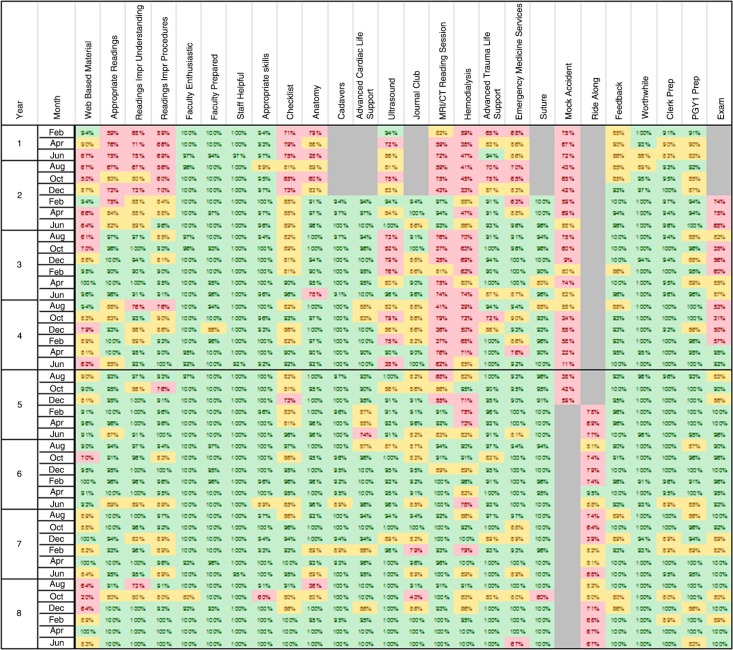
Percentage of students (by month and by academic year) who positively endorsed 27 individual program elements of the Clinical Skills Immersion Experience (CSIE) course at The Ohio State University College of Medicine The bold line between the end of year 4 and the beginning of year 5 indicates when the peer-teaching model was implemented. Color code key: Green: 90% or more of the students from the rotation (month and year) positively endorsed the program element. Yellow: 80–89% of the students from the rotation positively endorsed the program element. Red: Fewer than 80% of the students from the rotation positively endorsed the program element. Gray: The program element was not offered during these times.

Beyond high global ratings of the course's utility, learners also positively endorsed the faculty enthusiasm and knowledge (98.9%), CSEAC personnel (99.7%), and the selection of procedural skills taught (97%). See Fig. 1 for a summary of the percentage of positively endorsed CSIE program components broken down by month and academic year.

Students demonstrated a clear preference for simulations and hands-on practice. They rated the cadaver lab, which was implemented during the second year of the course, as the highest program activity (97.3% positive endorsement). Procedures practiced in the cadaver lab included: tube thoracostomy, lumbar puncture, intubation, thoracentesis, paracentesis, and pericardiocentesis. For the last three skills, cadavers were prepared by our anatomy staff to provide fluid return when the student cannulated the correct anatomical space.

Students were slightly less enthused about assigned readings and procedural videos. They rated assigned readings (90.3% positive endorsement) and procedural videos (86.6% positive endorsement) as useful. Students commented that readings helped provide the ‘big picture’ and that videos prepared them for practicing the procedures themselves. Students also agreed that the procedural checklists which included indications, contraindications, and step-by-step instructions for the procedure were helpful (86.4% positive endorsement).

Less well accepted were the lectures on hemodialysis and MRI/CT interpretation which received 73.1 and 79.2% positive endorsements, respectively. The former was felt to be too advanced for third-year medical students and the latter was thought to be taught in groups that were too large (15 per group).

Students appreciated the ACLS refresher followed by the 30 min megacode simulation scenarios (93.9% positively endorsed). Students expressed an interest in more of these types of simulations during the course.

The advanced trauma life support (ATLS) was also reviewed positively, but suffered because it was less ‘hands-on’. The emergency medical services (EMS) ride-alongs were moderately well received, but time limitations (only a 3-h ride-along) meant that some students experienced little to no activity. Subsequently this activity, which was designed to show students how ATLS algorithms were implemented in the field, received only a 75.3% positive endorsement.

### Peer versus individual teaching

In June, 2010 (year 4 of the program) students were assigned to peer-teams that worked together for the duration of the course. The introduction of peer-teams to the program had significant positive effects on student's attitudes toward program activities as measured by the course evaluation. We compared evaluation ratings from before and after this change using Pearson chi-square tests and observed statistically significant improvements. All analyses were performed with SAS JMP (16). After the change, we found student ratings of assigned readings went up, including: appropriateness of readings, which went from 84.1 to 95.1% positive endorsement (X^2^(4)=37.5; *p*≤0.001); contribution of readings to comprehension, which went from 85.1 to 95.5% positive endorsement (X^2^(4)=36.8; *p*≤0.001); and improved comfort with procedures based on readings, which went from 80.2 to 93% positive endorsement (X^2^(4)=37.2; *p*≤0.001).

Implementation of the peer-teaching model also had positive and statistically significant effects on some of the other less popular course activities. Ratings of the hemodialysis lecture went from 59.8 to 86.7% positive endorsement (X^2^(4)=101.1; *p*≤0.001). Ratings of the MRI/CT session went from 68.0 to 90.5% positive endorsement (X^2^(4)=93.6; *p*≤0.001). Figure 1 shows the positive endorsement change before and after the peer-team teaching model (bold line).

## Discussion

The CSIE ensured that all students received standard coverage of procedures considered to be important for residency preparation. The program was generally well received by students. Students preferred hands-on experiences over lectures, which is not surprising given the practical nature of the content material. This was a relatively resource-intense course, but was made more manageable through the use of supplemental training videos and online learning modules. Faculty from a variety of disciplines contributed to the teaching load, delivering content that was specific to their areas of expertise. The course relied heavily on our new well-equipped and well-staffed CSEAC.

The switch to a peer-team teaching model contributed to improved satisfaction with the course, while decreasing faculty time. Whereas improvement in student attitudes toward the course may be attributed to reducing the individual's ‘stakes’ and workload, we did not observe a reduction in learning or performance. Cooperative Learning Theory suggests that adult learners prefer learning in teams (17). Future research might compare the learner outcomes of procedures training through a peer-team teaching model to traditional training models.

While preparing this manuscript, we discovered a proposed educational framework for learning procedural skills that fits with our CSIE curriculum. The first three steps of Sawyer's six-step framework for procedural skill development ‘Learn, See, Practice, Prove, Do, and Maintain’ describes the structure and intended purpose of our CSIE curriculum in that our students were introduced to new skills, saw them demonstrated in the course and on service, and then had opportunities to practice them through various methods of simulation (18). Our hope was that the CSIE course prepared students for success with the final three stages of procedural skills development ‘Prove, Do, and Maintain’ during their senior year and throughout residency.

Starting with this academic year, the CSIE course was replaced by three separate ‘ground school’ courses linked to three 16-week clerkships covering: ‘Understanding Patients within Populations’, ‘Understanding Patients with Reproductive and Surgical Needs’, and ‘Understanding Patients with Special Medical Needs’. The ground schools prepare students for the remainder of the clerkship by covering procedures most commonly used in those clerkships. In effect, we have been able to move the learning closer to the care environment in which the procedures are most likely to be needed and applied.

This study has several limitations. Because the scope of the course was designed for breadth and not depth of learning, procedural mastery was not measured. Nor were efforts made to assess skill retention over time. Accordingly we cannot authoritatively state that the course had long-term benefits beyond medical school. A second limitation was the lack of direct assessment of student–patient communication involving procedures. We relied on student ratings of course components to inform us about the contribution of program activities to their learning. However, a more direct measure of the impact of the course on medical student communication with patients, particularly when conversations involved procedures, would have been preferred. Finally, we also experienced limitations on the size and scope of the end-of-course evaluation. Although we were able to adapt the traditional standard form used by the medical school, we were not able to capture specific details about student efficacy that would have contributed to further understanding about the value of this course.

## Conclusion

The CSIE course was an attempt to introduce routine clinical procedural skills to third-year medical students, and to provide them with sufficient practice to understand the patient experience during procedures. Furthermore, students gained sufficient knowledge about a broad range of procedures as a foundation for future practice and mastery. The course was resource intense, but had the immediate benefit of providing students with sufficient confidence to continue their learning. Although the course's quality gradually improved over time, the switch to peer-team teaching seemed to have the biggest impact on student satisfaction.
